# *DEP1* gene in wheat species with normal, compactoid and compact spikes

**DOI:** 10.1186/s12863-017-0583-6

**Published:** 2017-12-28

**Authors:** Valeriya Vavilova, Irina Konopatskaia, Anastasia E. Kuznetsova, Alexandr Blinov, Nikolay P. Goncharov

**Affiliations:** Institute of Cytology and Genetics SB RAS, Novosibirsk, Russia

## Abstract

**Background:**

In rice, a variant of *DEP1* gene results in erect panicle architecture, well-developed vascular bundles, an increase in the number of grains per panicle and a consequent increase in the grain yield. Interestingly, *DEP1* homologs are present in the other cereals including species of wheat and barley (*Hordeum vulgare*), even though they do not produce panicles but spikes. In barley, *HvDEP1* alleles do not differ between strains of various ear types and geographic origins, while in at least three *OsDEP1* variants have been described.

**Results:**

In this work, we have studied the *DEP1* gene from eight accessions which belong to four wheat species, *T. monococcum, T. durum, T. compactum,* and *T. spelta*, with either compact, compactoid or normal spike phenotypes. The nucleotide sequences of the 5th exon of *DEP1* were determined for all eight accessions. Obtained sequences were species specific. Despite the interspecies diversity, all wheat sequences encoded polypeptides of the same size, similarly to the 5th exons of the *DEP1* homologs in *T. aestivum, T. urartu*, and *H. vulgare*. For further study, the full-length sequences of the *DEP1* gene for all four species were studied. The full-length *DEP1* genomic copies were isolated from the genomic sequences of *T. aestivum, T. urartu,* and *Aegilops tauschii*.

The genome of tetraploid wheat *T. durum* contains two variants of the *DEP1* originating from A and B genomes. In the hexaploid wheats *T. aestivum, T. compactum,* and *T. spelta,* three variants of this gene originating from A, B, and D genomes were detected. *DEP1* genes of the diploid wheats *T. monococcum* and *T. urartu* differ. It seems that a precursor of the *DEP1* gene in *T. monococcum* originates from the wild progenitor *T. boeoticum*.

**Conclusion:**

No *DEP1*-related differences of nucleotide sequences between the compact (or compactoid) and normal spike phenotypes in the tested wheat species were detected. Therefore, *DEP1* gene does not directly participate in the control of the spike architecture in wheats.

**Electronic supplementary material:**

The online version of this article (10.1186/s12863-017-0583-6) contains supplementary material, which is available to authorized users.

## Background

Spike shape is a taxonomically important characteristic of the genus *Triticum* L. (tribe Triticeae Dum., family Poaceae Barn.). Three main variants of spike shape, namely, normal, spelt and compact, are widely distributed among wheats. Cultivated wheat species possess relatively short square headed parallel-sided spikes – so-called “normal” spikes [1], while the spikes of *Triticum spelta* L., *T. dicoccum* (Schrank) Schuebl., and *T. dicoccoides* (Körn. ex Aschers. et Graebn.) Schweinf. are of pyramidal shape with elongated rachis and tenacious glumes (“spelt type”) [1]. The third variant of spike morphology is called “сompact”; these are short, dense spikes with fewer spikelets per spike recently described in diploid *T. sinskajae* and two hexaploid species, *T. compactum* Host and *T. sphaerococcum* Perciv. [1]. Additionally, a subvariant “compactoid”, which is characterized by less dense spikes as compared to “compact” type, is found in the hybrids of *T. dicoccoides* and *T. dicoccum* [2, 3].

Genetic control of the spike shape trait in wheats is poorly investigated. Several genes and loci related to spike shape trait have been identified and include the following: *Q*, *C*, *C2*, *sc1* and *sc2* (for review: [4]). Among listed above *Q* gene was localized on the chromosomes of homeological group 5 and molecularly cloned. Gene *C* was located on the long arm of chromosome 2D, while the sequences and genome localization of the *C2*, *sc1* and *sc2* remain unknown [3, 5–9]. The transcription factor gene, *Q*, has a pleiotropic effect on spike shape in polyploid wheat species [9]. The dominant *5AQ* and recessive *5Aq* alleles differ in two nucleotide substitutions and determine either normal or spelt spike phenotype [9–11]. *5Dq* and *5 Bq* alleles are involved in the suppression of the spelt phenotype [10]. No investigation supports the influence of *Q* gene on the formation of compact and compactoid spikes of polyploid wheat species.

*DENSE AND ERECT PANICLE 1* (*DEP1*) gene extensively investigated in rice could contribute to the spike shape trait in wheat (for review [12]). In rice the *DEP1* gene located on the chromosome 9 pleiotropically controls several traits including panicle density, grain number per panicle and erect panicle among others. Experiments with near-isogenic lines showed that *OsDEP1* gene is a dominant negative regulator of panicle architecture and grain number. The *DEP1* gene encodes the phosphatidylethanolamine-binding (PEBP) protein consisting of the following modules: plant-specific Gγ subunit protein domain, three von Willebrand factor type C (VWFC) domains and a tumor necrosis factor receptor (TNFR)/nerve growth factor receptor (NGFR) family cysteine-rich domain [13]. The mutant *DEP1* allele (*dep1*) possess a replacement of a 637 bp stretch from the middle of the 5th exon by a short sequence 12 bp in length which results in loss of cystein-rich domain at the C-terminus of Gγ subunit. Rice varieties with the *DEP1* allele are characterized by the erect panicle architecture, well-developed vascular bundles, an increased number of grains per panicle and increased grain yield.

Huang et al. [14] investigated *DEP1* homologues from *T. urartu* Thum. ex Gandil., *T. aestivum* L. and *Hordeum vulgare* L., and identified variable deletions within 5th exon in comparison with to the dominant allele described in rice. The detailed characterization of the *HvDEP1* gene for the large collection of barley cultivars showed no correlation between *HvDEP1* gene sequences and ear phenotype [15]. The upstream open reading frame (uORF) identified in the 5′UTR of *HvDEP1* is predicted to contribute to post-transcriptional regulation of the gene. In case of *T. aestivum* the experiments with the transgenic line showed that downregulation of *DEP1* homologue affects the length of the ear, ear density and number of spikelets [14]. Thus, the identification of this gene in wheat species with different spike shape will expand our knowledge of *DEP1* role in the evolution of cereals.

## Methods

### Plant materials

Germplasm of *T. monococcum* L. (4 accessions), *T. durum* Desf. (2 accessions), *T. compactum* (1 accession), and *T. spelta* (1 accession), were obtained from Gifu University (Gifu, Japan), Graduate School of Agriculture of Kyoto University (Kyoto, Japan), N.I. Vavilov All-Russian Institute of Plant Industry (St-Petersburg, Russia), The Federal Research Center Institute of Cytology and Genetics The Siberian Branch of the Russian Academy of Sciences (Novosibirsk, Russia), National Small Grains Collection (Aberdeen, USA) and Kh.Yu. Yusufbekov Pamir biological institute (Khorog, Republic of Tajikistan). Ten plants of each accession were growing in the greenhouse under the standard conditions. Spike shape was determined visually and confirmed by calculation of Flaksberger’s formula [16]. The significance of differences between accessions with normal and compact (or compactoid) spikes was determined by the Student test. The results are presented in the Table 1 and Additional file 1: Table S1.

**Table 1:** List of wheat accessions used for sequence analysis of the *DEP1* gene

Genome	Species	Accession number	Place of origin	Spike shape	*DEP1* GenBank No.
A^b^	*T. monococcum* L.	*Sog glume*-*1*	Mutant obtained in Japan [24]	compactoid	MF979621
A^b^	*T. monococcum* L.	*Sog glume*-*2*	Mutant obtained in Japan [24]	compactoid	MF979622
A^b^	*T. monococcum* L.	Extremely early C	Mutant obtained in Japan	normal	MF979623
A^b^	*T. monococcum* L.	К-18105	Nagorno-Karabakh	normal	MF979624
BA^u^	*T. durum* Desf.	Sharik	Russia [25]	compactoid	MF979625, MF979631
BA^u^	*T. durum* Desf.	Lnd222 (CI12341)	USA	normal	MF979626, MF979632
BA^u^D	*T. compactum* Host	K1711	Gorno-Badakhshan Autonomous Region	compact	MF979627, MF979629, MF979633
BA^u^D	*T. spelta* L.	К-53660	Gorno-Badakhshan Autonomous Region	Normal	MF979628, MF979630, MF979634

### *DEP1* gene extraction from WGS databases

Full-length sequences of *DEP1* gene of *T. aestivum*, *T. urartu*, and *Aegilops tauschii* Coss. were extracted from Whole-Genome Shotgun contigs (WGS) database (https://www.ncbi.nlm.nih.gov/genbank/wgs/) applying Basic Local Alignment Search Tool (BLAST) (Additional file 1: Table S2) [17]. *DEP1* mRNA sequences of *T. aestivum* (GenBank No. FJ039902) and *T. urartu* (GenBank No. GQ324995) were used as query sequences for the BLAST search.

### *DEP1* gene cloning and sequencing

Total DNA was isolated from 100 mg of leaves using the DNeasy Plant Mini Kit (QIAGEN) according to the manufacturer’s protocol.

The *DEP1* mRNA sequences of *T. urartu* (GQ324995), *T. aestivum* (FJ039902) and *H. vulgare* (FJ039903) were used to design primers to amplify *DEP1* gene from the *T. monococcum*, *T. durum*, *T. compactum*, and *T. spelta*. Primer pair for the amplification of 5′UTR of the *DEP1* was constructed using full-length *DEP1* sequence of *T. aestivum* extracted from WGS database (Additional file 1: Table S2). The genomic *DEP1* gene sequence was PCR-amplified as five separate overlapping fragments using primers pairs listed in Additional file 1: Table S3. Schematic representation of the primer pairs positions and PCR conditions for each fragment are shown in the Additional file 1: Table S3 and Figure S1. PCR products were separated by agarose gel electrophoresis and purified using a QIAquick Gel Extraction Kit (QIAGEN). For *T. monococcum* accessions purified PCR products were sequenced directly without cloning. For polyploid wheat species purified PCR fragments were cloned into a pGEM®-T Easy vector using a pGEM-T Easy kit (Promega) and amplified with M13 primers prior to sequencing (Additional file 1: Table S3). Sequencing reactions were performed with 200 ng of the PCR product and ABI BigDye Terminator Kit on an ABI 3130XL Genetic Analyser (Applied Biosystems) in SB RAS Genomics Core Facility (http://www.niboch.nsc.ru/doku.php/corefacility). In total 10 clones were sequenced for each *DEP1* fragment from 4 accessions of *T. durum*, *T. spelta* and *T. compactum*. *DEP1* gene sequences were deposited in GenBank (Table 1).

### *DEP1* gene sequences analyses

Nucleotide and amino acid sequences alignments were performed using Vector NTI Advance version 10.0 program and improved with the MUSCLE algorithm in UGENE software (http://ugene.unipro.ru/) [18, 19]. ORFs in DNA sequences were predicted using ORF Finder program available from National Center for Biotechnology Information (NCBI) (https://www.ncbi.nlm.nih.gov/orffinder/). Phylogenetic analysis was performed by Maximum likelihood method (ML) integrated in PhyML 3.0 program [20]. Statistical support for the ML tree was evaluated by bootstrapping [21]. p-distances for *DEP1* and *VRN1* genes sequences were calculated using MEGA5 software [22]. UGENE software was used for the multiple sequence alignments visualization and editing [19].

## Results

### Variability of the predicted 5th exon of *DEP1* gene in wheat species

In rice *DEP1* gene the gain-of-function mutation is located within the 5th exon region. Using the Dep1-MF/Dep1-MR primer pair we obtained the sequences of the predicted 5th exon of the *DEP1* gene for the *Triticum* species listed in the Table 1. A and D genome copies of *DEP1* gene of *T. aestivum* (FAOM01374184 – 5AL chromosome, FAOM01435944 – 5DL chromosome) and *DEP1* gene sequence of *Ae. tauschii* (MCGU01048243) were extracted from WGS database. The third *DEP1* sequence of *T. aestivum* (CCYC011397742) extracted from WGS database is predicted to be B genome homologues of the gene. To investigate differences between the predicted 5th exon sequences, we aligned the newly obtained sequences and sequences extracted from WGS database with *DEP1* mRNA of *T. urartu* (GQ324995), *T. aestivum* (FJ039902) and *H. vulgare* (FJ039903)*.* The results of comparative analysis are presented in the Additional file 1: Figure S2.

**Fig. 1:**
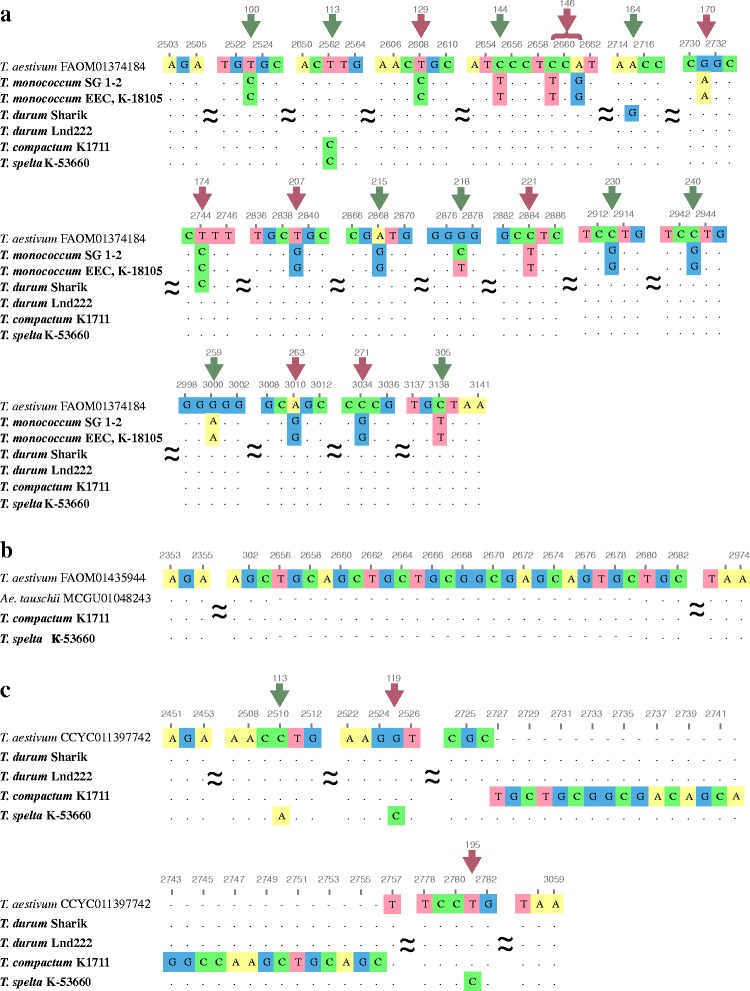
Variability of 5th exon of *DEP1* gene from di-, tetra- and hexaploid wheat species. **a** Newly obtained *DEP1-A* sequences from *T. monococcum*, *T. durum*, *T. compactum*, *T. spelta* and reference sequence of *T. aestivum* (FAOM01374184) extracted from WGS database. **b** Newly obtained *DEP1-D* sequences of *T. compactum* and *T. spelta* and reference sequence of *T. aestivum* (FAOM01435944) extracted from WGS database. **c** Predicted B genome homologues of *DEP1* gene from *T. durum*, *T. compactum*, *T. spelta* and reference sequence *T. aestivum* (CCYC011397742) extracted from WGS database. Wheat accessions analyzed in the present study are marked in bold. Nucleotides matching with reference sequence are designated by dots. Nonsynonymous and synonymous amino acid substitutions are indicated by red and green arrows, respectively. Numbers of the nucleotides upstream from the start codon are given in accordance with the *DEP1* sequences of *T. aestivum*. Numbers of codons are presented above the rows according to *DEP1* of *T. aestivum*

One variant of the predicted 5th exon sequence 639 bp in length was detected for the analyzed *T. monococcum* accessions. We observed one sequence change that distinguishes accessions with compactoid and normal spikes: C/T substitution in the position 375 didn’t lead to amino acid substitution (Fig. 1a, Additional file 1: Figure S2).

Two different sequences of the predicted 5th exon (639 bp and 579 bp in length) were identified for the accessions of *T. durum*. Longer sequence of *T. durum* Lnd222 was identical to sequence of *T. aestivum* (FAOM01374184 – 5AL chromosome) (Fig. 1a). The second sequence from *T. durum* Lnd222 differed from A genome copy by five deletions and 24 nucleotide substitutions that caused 9 amino acid changes (Additional file 1: Figure S2). In case of *T. durum* Sharik the second sequence of the 5th exon was separated by the same five deletions and 23 nucleotide substitutions (Additional file 1: Figure S2). Analysis of the ORFs showed that only 8 out of 23 substitutions led to the amino acid changes. Shorter sequences from *T. durum* Sharik and *T. durum* Lnd222 were similar to the *DEP1* from *T. aestivum* (CCYC011397742) and are predicted to refer to *DEP1-B* gene (Fig. 1c, Additional file 1: Figure S2). Nucleotide sequences of the 5th exon of *DEP1-A* of *T. durum* accessions with normal and compactoid spikes differed by two substitutions (positions 213 and 242). Substitution CCT/CTT in codon 218 led to amino acid substitution P/L (Fig. 1a). No differences in *DEP1-B* sequences which separate *T. durum* accessions with normal and compactoid spikes were identified (Fig. 1c).

Three sequences of the predicted 5th exon were obtained for *T. compactum* K1711 and *T. spelta* К-53660. Sequences of 639 bp in length were identical for these accessions and showed 99.8% similarity with *DEP1* sequence of *T. aestivum* (FAOM01374184 – 5AL chromosome) (Fig. 1a, Additional file 1: Figure S2). D genome copies of *DEP1* the predicted 5th exon from analyzed *T. compactum* K1711 and *T. spelta* К-53660 accessions showed 100 and 96% similarity to *T. aestivum* FAOM01435944, correspondingly. *T. spelta* accession possess a 24 bp deletion from position 304 to 328 when comparing to *T. compactum* K1711 and *T. aestivum* FAOM01435944 (Fig. 1b, Additional file 1: Figure S2). Analogous deletion is presented in *Ae. tauschii* (MCGU01048243). The deletions described above did not cause frame shifts as it was shown for the recessive allele of *DEP1* gene in *O. sativa* [14]. The third variants of the predicted 5th exon sequences of *T. compactum* K1711 differed by one insertion of 30 bp in length and showed high similarity to the sequences of *T. aestivum* (CCYC011397742). The last variant of the predicted 5th exon sequences of *T. spelta* К-53660 showed 99.5% similarity with *DEP1* sequence of *T. aestivum* (CCYC011397742) (Fig. 1c, Additional file 1: Figure S2).

### Variability of 5′UTR of *DEP1* gene in wheat species

We identified 5′ UTR (approximate length 250 bp) of *DEP1* gene for accessions of *T. monococcum*, *T. durum*, *T. compactum* и *T. spelta* using Dep1-LF/Dep1-LR primer pair (Additional file 1: Table S1). One variant of 5′UTR was obtained for each accession of analyzed di- and tetraploid wheat species. Length of the sequence was 254 bp and 256 bp in *T. monococcum* and *T. durum*, correspondently. For both *T. compactum* K1711 and *T. spelta* К-53660 we obtained two variants of 5′UTR of 256 bp and 259 bp in length.

**Fig. 2:**
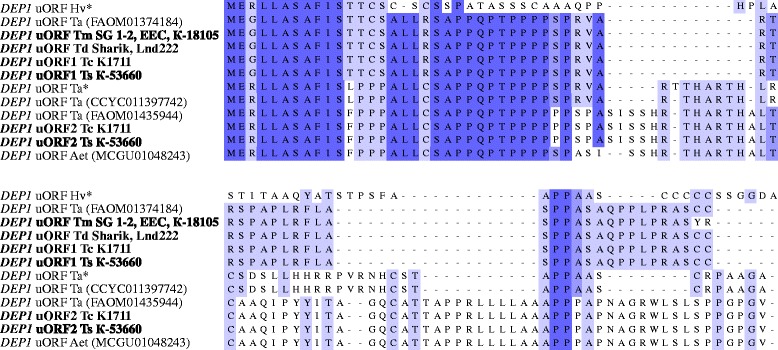
Alignment of upstream open reading frames, determined in 5′UTR of *DEP1* gene from several grasses. Single asterisk indicates sequences identified by Belanger et al. [15]. Abbreviations: uORF - upstream open reading frame, Hv – *Hordeum vulgare*, Ta - *Triticum aestivum*, Tm - *Triticum monococcum*, Td – *Triticum durum*, Tc – *Triticum compactum*, Ts - *Triticum spelta*, Aet - *Aegilops tauschii*. Wheat accessions analyzed in the present study are marked in bold. Amino acid residues are colored according to the percentage identity in the UGENE software [19]

ORFs were predicted for newly obtained sequences and 5′UTRs of *DEP1* from wheat, barley and *Aegilops* available from databases. The results showed that all 5′UTRs from species listed in Table 1 possess the uORF that varied in length from 63 to 98 amino acids (Fig. 2). N-terminus of the predicted amino acid sequences was conservative among studied species while the rest part varied highly. uORFs of *T. aestivum* (FAOM01374184), accessions of *T. durum* and first variant of *T. compactum* K1711 and *T. spelta* К-53660 uORFs were 63 bp in length and identical to each other. Sequences from studied *T. monococcum* accessions differed from listed above by two amino acid changes. Length of uORF predicted for *T. aestivum* (CCYC011397742) was 76 amino acids. The second variant of uORF from *T. aestivum* (FAOM01435944), *T. compactum* K1711 and *T. spelta* К-53660 were identical (length 98 amino acids) and differed from the uORF of *Ae. tauschii* (MCGU01048243) by to amino acid substitution (Fig. 2).

uORFs were identified for *DEP1* genes of all studied accessions and thus confirm the suggestion that presence of uORF is conservative characteristic of orthologous genes *DEP1* in grasses [15].

### Full-length *DEP1* gene sequence: comparative and phylogenetic analysis

To investigate variability within predicted introns and the first 4 exons of the *DEP1* gene we obtained full-length gene sequences from 8 accessions of di-, tetra-, and polyploid wheat species (Table 1). Four *DEP1* nucleotide sequences of *T. monococcum* accessions were identified by direct sequencing of PCR fragments. In case of polyploid species were identified *DEP1* sequences by PCR, cloning and sequencing. Full-length sequences were constructed by alignment of overlapping fragments.

#### Comparative analysis of *DEP1* gene from A genome

**Fig. 3:**
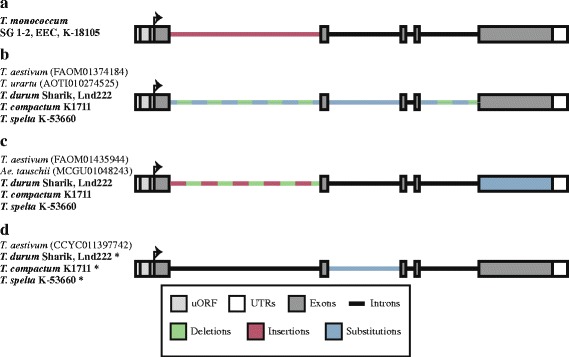
Scheme of variable and conservative regions of *DEP1* gene from wheat species and *Aegilops tauschii*. **a** *DEP1* gene from *Triticum monococcum* accessions. **b** *DEP1-A* gene from polyploid wheat species and diploid *Triticum urartu*. **c** *DEP1-D* gene from polyploid wheat species and *Aegilops tauschii*. **d** Predicted B genome homologues of *DEP1* gene from wheat species. Wheat accessions analyzed in the present study are marked in bold

Figure 3 illustrates variable and conservative regions of *DEP1* gene found in the wheat species studied and *Ae. tauschii*. Nucleotide sequences of all four accessions of *T. monococcum* encode identical protein sequences, although two of those accessions have compactoid spikes and the other two have normal spike. The only difference between *DEP1* gene sequences from accessions with compactoid and normal spikes is the length of the (TC) track within the first intron. Nucleotide sequences of *T. monococcum Sog glume-1* and *T. monococcum Sog glume-2* have (TC)_5_ repeat, while accessions Extremely early C and К-18105 have (TC)_11_ repeat (Fig. 3a).

Several *DEP1* sequences were obtained for polyploid wheat accessions. In each accession of *T. durum*, *T. compactum* and *T. spelta* we identified *DEP1* sequence that was identical to *T. aestivum* (FAOM01374184 – 5AL chromosome) in the coding region and thus, was referred to the A genome. However, non-coding regions of *DEP1-A* are characterized by minor differences (Fig. 3b). 5′ UTR of *T. spelta* К-53660 possesses a deletion of 12 bp, while *T. urartu* possesses a deletion of two nucleotides in the first intron and an insertion of two nucleotides in the fourth intron. In addition, nucleotide sequence of the intron 2 of *T. urartu* contains five nucleotide substitutions compared to the sequences of *DEP1* gene of other accessions. Finally, the nucleotide sequences of *T. urartu* and *T. durum* Sharik accessions differ from sequences of *T. aestivum* (FAOM01374184), *T. durum* Lnd222, *T. spelta* К-53660 and *T. compactum* K1711 accessions by six nucleotide substitutions in the first intron and one nucleotide substitution in the fourth intron (Fig. 3b).

#### Comparative analysis of *DEP1* gene from D genome

In the *T. compactum* K1711 and *T. spelta* К-53660 we identified *DEP1* sequences similar to the *T. aestivum* (FAOM01435944 – 5DL chromosome) and *Ae. tauschii* (MCGU01048243) and, thus as a result were referred to the D genome. In contrast to A genome, the *DEP1* gene sequences of D genome showed variability in coding region among studied species (Fig. 3c). *Ae. tauschii* (MCGU01048243) and *T. spelta* К-53660 contain a deletion of 24 nucleotides in compare to *T. aestivum* (FAOM01435944) and *T. compactum* K1711. Differences are presented in the non-coding regions as well. The *DEP1* gene of *Ae. tauschii* possesses a deletion of six nucleotides in the 5′ UTR and two insertions in the first intron of 1 and 5 bp in length. 5′ UTR and the first intron of *T. spelta* К-53660 contain deletions and insertions, however, their positions are different from *Ae. tauschii* (Fig. 3c).

#### Comparative analysis of predicted B genome homologues of *DEP1* gene

Additional variant of *DEP1* identified for the accessions of polyploid wheat showed high similarity to *DEP1* of *T. aestivum* (CCYC011397742) (Fig. 3d). These sequences were obtained partially and 5′UTR, exon 1 and intron 1 remain unknown. Comparative analysis of all these sequences showed no differences in the coding region, although several nucleotide substitutions were found in the predicted intron 2 (Fig. 3d). In comparison with *T. aestivum, T. spelta* К-53660 had three nucleotide substitutions, while *T. compactum* K1711 had two nucleotide substitutions, and both samples of *T. durum* had no substitutions at all. These sequences were predicted to be the *DEP1-B* gene and showed 8.2 and 4.6% dissimilarity to the *DEP1-A* and *DEP1-D* sequences, correspondingly. However, within genomes p-distances were less and varied 0–3.4 and 0.2–0.3% in case of *DEP1-A* and *DEP1-D* gene sequences, correspondingly (Additional file 1: Table S4). Similar situation was detected for another wheat gene *VRN1*. Distance between *VRN1* alleles from the same genome was lower than distance between sequences from different genomes (Additional file 1: Table S5).

#### Phylogenetic analysis of *DEP1* genes

**Fig. 4:**
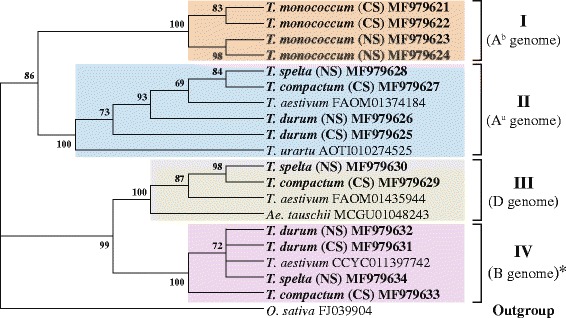
Maximum likelihood phylogenetic tree constructed based on *DEP1* gene sequences from wheat species and *Aegilops*. *OsDEP1* gene sequence from *O. sativa* (FJ039904) was used as outgroup. Statistical support was evaluated by bootstrapping (1000 replications); nodes with bootstrap values over 50% are indicated. Clusters are highlighted in different colors. Sequences obtained in this study are marked in bold. Abbreviations: CS – compact or compactoid spike, NS – normal spike. ^*^ - predicted B genome

Phylogenetic analysis was performed using DNA sequences of newly obtained *DEP1* genes sequences from different wheat species (Table 1). Sequence of the *DEP1* from *O. sativa* (FJ039904) was used as an outgroup. Four clusters in addition to the outgroup are presented in the phylogenetic tree (Fig. 4). The first cluster (I) is formed by the *DEP1* gene sequences from A^b^ genome of *T. monococcum* accessions. *DEP1* sequences of these accessions are grouped into two clades according to the spike shape (compactoid or normal). The second cluster (II) consists of *DEP1* sequences from five species: *T. urartu, T. aestivum, T. durum, T. spelta* and *T. compactum* (Fig 4). These sequences belong to the A^u^ genome and are grouped together but not according to spike shape of wheat accessions. All *DEP1* sequences from D genomes of wheat species are in the III cluster together with *DEP1* of *Ae. tauschii.* The last cluster (IV) is formed by the *DEP1* sequences which are highly similar to the predicted B genome homologues of *DEP1* of *T. aestivum* (CCYC011397742). The *DEP1* sequences from polyploid wheats were not grouped in the phylogenetic tree according to the spike phenotype (Fig. 4).

## Discussion

### *DEP1* gene and spike shape trait in wheat

Genetic control of spike shape, which is one of the main morphological trait of the wheat species, is poorly known. Nowadays only *Q* gene which pleiotropically affect spike shape trait in polyploid wheat species was well studied [9–11]. Other loci related to the trait such as *C*, *C2*, *sc1* and *sc2* are poorly investigated [3, 5, 8]. We choose *DEP1* gene previously identified in rice as a candidate for the role of spike shape trait regulator in wheat. In rice the *DEP1* is a dominant negative regulator of panicle architecture and grain number [14]. Homologues of the gene were characterized in 5H chromosome of barley [15]. In case of wheat species only mRNAs of *DEP1* were identified for *T. aestivum* и *T. urartu*. The above-mentioned species are characterized by normal spike shape and did not allow to investigate the influence of *DEP1* gene on spike shape trait.

In this study full-length gene sequence of *DEP1* was identified for the eight accessions of four wheat species icluding di-, tetra- and hexaploids with normal, compact and compactoid spikes (Table 1). Furthermore, full-length *DEP1* sequences of *T. aestivum*, *T. urartu* and *Ae. tauschii* were extracted from the WGS database*.* Comparative and phylogenetic analysis of obtained *DEP1* sequences revealed no mutations that distinguish accessions with normal spikes from compact or compactoid phenotype (Figs. 1, 2 and 3). Moreover, in case of *T. monococcum* и *T. durum* no intraspecific variability of the coding region of *DEP1* gene was identified despite investigation of both normal and compactoid accessions of the same species. Therefore, the results of the study rose the questions about the influence of the *DEP1* gene on spike architecture in wheat.

Belanger et al. [15] performed similar analysis for the *DEP1* in various two-row and six-row accessions of barley from different geographic origins. They also did not identify the gene sequence variability which could separate accessions with different phenotype. All identified mutations were located within introns except for three SNPs in the first and the fifth exon that however resulted in silent mutations [15]. However, relatively long 5′ UTR of *DEP1* encoding peptide of 70 amino acids was identified and predicted to contribute to post-transcriptional regulation of the gene. We identified uORFs within the 5′ UTR of *DEP1* genes for all studied species and suggested that it could contribute to the post-transcriptional regulation of *DEP1* and formation of normal or compact (compactoid) spikes in wheat (Fig. 2).

Although we did not identify mutation within *DEP1* gene sequence, which could distinguish wheat accessions based on the spike morphology. This gene takes part in the regulation of the spike shape trait. Huang et al. [14] showed that downregulation of *DEP1* in *T. aestivum* leads to increased length of the ear, makes it less compact and reduces number of spikelet. Thus, investigation of other loci related to the spike shape trait is essential to determine mode of the *DEP1* influence.

### *DEP1* gene and origin of Triticum species

Previously, we suggested the scheme of the wheat species origin and evolution [23]. The results of this study supported the above-mentioned scheme. The *DEP1* gene from A^b^ genome was presented only in *T. monococcum* accessions and was not identified for the polyploid wheat species. This fact supported the hypothesis that A^b^ genome of the *T. monococcum* was inherited from *T. boeoticum* and was not presented among polyploid wheat species. *T. urartu* is a donor of second variant of the A genome (A^u^) for all polyploid wheats. In this study one variant of *DEP1* gene was identified for both *T. urartu* and polyploids analyzed. On the phylogenetic tree *DEP1* gene from *T. urartu* took basal position towards *T. durum*, *T. aestivum*, *T. compactum* and *T. spelta*. Thus, we supported the hypothesis of the inheritance of A^u^ genome by polyploid wheat species from *T. urartu*.

According to the scheme [23] the *Ae. tauschii* is a donor of D genome for hexaploid wheat species of section Emmer. Thus, this study supports this way of inheritance. Indeed, the variant of *DEP1* gene extracted from WGS database was identified in hexaploid wheat species and absent in tetraploid species *T. durum* (Figs. 3 and 4). В genome of tetra- and hexaploid wheat species originated from *Ae. speltoides*. The sequence of *DEP1* gene from *Ae. speltoides* is unknown, but one of the gene variant identified among polyploids wheat only is predicted to be a B genome homologue of this gene. The level of dissimilarity between predicted *DEP1-B* sequences and *DEP1-A* and *DEP1-D* sequences is higher than dissimilarity within sequences from the same genome. The same situation was shown for another wheat gene *VRN1* (Additional file 1: Table S4). On the phylogenetic tree predicted *DEP1-B* sequences were grouped into one cluster separated from *DEP1-A* and *DEP1-D* clusters (Fig. 4). Thus, the results of comparative and phylogenetic analysis showed that the predicted *DEP1-B* sequences are not the new alleles of A or D genomes and support the suggestion that *DEP1* of *T. aestivum* (CCYC011397742) and sequences of *T. durum* (MF979631, MF979632), *T. spelta* (MF979634) and *T. compactum* (MF979633) represent B genome homologues of *DEP1*.

## Conclusions

In this study full-length *DEP1* gene sequences from 8 accessions of di-, tetra-, and hexaploid wheat species were obtained. In addition, full-length sequences of *DEP1* gene of *T. aestivum*, *T. urartu* and *Aegilops tauschii* were extracted from WGS database. The comparative and phylogenetic analysis revealed that tetraploid wheat *T. durum* contained two variants of the *DEP1* belonged to A and B genomes. Three variants of this gene belonged to A, B, and D genomes have been found in the hexaploid wheats *T. aestivum*, *T. compactum* and *T. spelta*. *DEP1* genes of the diploid wheats *T. monococcum* and *T. urartu* were different. It means that a precursor of the *DEP1* gene in *T. monococcum* should be from *T. boeoticum*. All the sequences obtained were species specific. *DEP1* gene mutations, distinguishing wheat accessions based on the spike morphology, were not found. uORFs were identified within the 5′ UTR of *DEP1* genes for all studied accessions. The uORF could contribute to the post-transcriptional regulation of *DEP1* gene and formation of normal or compact (compactoid) spikes in wheat. Thus, further investigations are required to determine *DEP1* gene engagement in the regulation of the spike shape trait.

## Additional file

Additional file 1: Table S1. Morphological characteristics of the wheat species used in the present study. **Table S2.** List of *DEP1* gene sequences of *Triticum* and *Aegilops* species obtained from WGS database. **Table S3.** Set of primers and PCR conditions used in the present study. **Table S4.** p-distances between wheat *DEP1* genes sequences from A, B and D genomes. **Table S5.** p-distances between wheat *VRN1* genes sequences from A, B and D genomes. **Figure S1.** Schematic representation of the primer pairs positions. **Figure S2.** Nucleotide alignment of 5th exon of *DEP1* gene from different wheat species, *Ae. tauschii* and barley. Sequence of *T. aestivum* (FAOM01374184) was used as a reference. Sequences obtained by experimental methods in the present study are marked in bold. Nucleotides, that match with reference sequence, are designated by dots. Nonsynonymous and synonymous amino acid substitutions are indicated by red and green arrows, respectively. (PDF 8916 kb)
