# Supplementary material for: DEP1 gene in wheat species with normal, compactoid and compact spikes
Source: BMC Genet. 2017 Dec 28;18(Suppl 1):106. doi: 10.1186/s12863-017-0583-6 (PMC5751790; doi:10.1186/s12863-017-0583-6)
Supplement: Additional file 1: Table S1. — Morphological characteristics of the wheat species used in the present study. Table S2. List of DEP1 gene sequences of Triticum and Aegilops species obtained from WGS database. Table S3. Set of primers and PCR conditions used in the present study. Table S4. p-distances between wheat DEP1 genes sequences from A, B and D genomes. Table S5. p-distances between wheat VRN1 genes sequences from A, B and D genomes. Figure S1. Schematic representation of the primer pairs positions. Figure S2. Nucleotide alignment of 5th exon of DEP1 gene from different wheat species, Ae. tauschii and barley. Sequence of T. aestivum (FAOM01374184) was used as a reference. Sequences obtained by experimental methods in the present study are marked in bold. Nucleotides, that match with reference sequence, are designated by dots. Nonsynonymous and synonymous amino acid substitutions are indicated by red and green arrows, respectively. (PDF 8916 kb) [file 12863_2017_583_MOESM1_ESM.pdf]

Additional file 1

Journal: BMC Genetics.

Title: *DEP1* gene in wheat species with normal, compactoid and compact spikes.

Authors: Valeriya Vavilova*, Irina Konopatskaia, Anastasia E. Kuznetsova, Alexandr Blinov and Nikolay P. Goncharov.

* - corresponding author: The Federal Research Center Institute of Cytology and Genetics SB RAS,

Prospekt Lavrentyeva, 10, Novosibirsk, Russian Federation, 630090; phone: +7 (383) 363-4969;

valeriya-vavilova@bionet.nsc.ru

Table S1. Morphological characteristics of the wheat species used in the present study.

No	Species	Accession number	Spike length, cm	Spikelet number per spike	Spike density*	Spike shape
1	*T. monococcum*	*Sog glume-1*	3.3±0.39	13.5±1.46	38.9±2.50[a]	compactoid
2	*T. monococcum*	Ext. early C	4.3±0.55	16.2±2.50	34.0±1.76	normal
3	*T. monococcum*	*Sog glume-2*	3.4±0.43	16.2±1.61	44.6±3.46[a]	compactoid
4	*T. monococcum*	К18105	4.7±0.79	19.7±2.22	37.8±1.00	normal
5	*T. durum*	Sharik	4.9±0.32	20.8±1.50	40.7±2.26[a]	compactoid
6	*T. durum*	Lnd222 (CI12341)	5.8±0.49	13.9±0.80	22.3±2.03	normal
7	*T. compactum*	к1711	4.9±1.11	15.8±1.81	32.5±4.49[a]	compact
8	*T. spelta*	к-53660	8.1±0.85	23.9±1.59	28.7±1.83	normal

*- Spike density was calculated by Flaksberger's formula [1]:

$D= [(A-1) \times 10]/ B$,

(A-1)– Spikelet number in spike (including the apical spikelet); B– spike length, cm; D– spike density.

1. Flaksberger KA. Pshenitsi- rod *Triticum* L. (Wheats- genus *Triticum* L.). Kul'turnaya flora SSSR. Khlebnie zlaki. Pshenitsa. (Cultivated Flora Sov. Union. Cereal. Wheat.). Gosudarstvennoe izdatel'stvo sovhoznoi i kolhoznoi literaturi. Moskva- Leningrad; 1935. p. 17–434.

[a] – The values are significant at $P < 0.01$ for each ploidy level (diploid No 1-2 and No 3-4; tetraploid No 5-6 and hexaploid No 7-8)

Table S2. List of *DEP1* gene sequences of *Triticum* and *Aegilops* species obtained from WGS database.

Genome	Species	WGS database No.
A	*T. aestivum* L.	FAOM01374184
D	*T. aestivum* L.	FAOM01435944
-	*T. aestivum* L.	CCYC011397742
D-like	*Ae. tauschii* Coss.	MCGU01048243

Table S3. Set of primers and PCR conditions used in the present study.

Primer name	Primer sequences	Target region
Dep1-LF<br>*Dep1-LR*	5’ –GCCGTGCGTGCAATCAAT– 3’<br>5’ –ACCTTGAGGAACGTGAGCT– 3’	Partial promoter and complete exon1 of *DEP1* gene
Dep1-GF<br>*Dep1-H2R*	5’ – GGAGGTGCAGATCCTTGACCG – 3’<br>5’ – CATACTCTTTCAAGCAACCAG – 3’	Partial exon1, complete intron 1 and exon2 of *DEP1* gene
Dep1-DF<br>*Dep1-B2R*	5’ – GGCTCAACCAGTCTCACGTT – 3’<br>5’ – GCAGCTTGGTCTTTTGCAGG – 3’	Partial exon2, complete intron2, exon3, intron3, exon4, intron4 and partial exon5 of *DEP1* gene
Dep1-AF<br>*Dep1-AR*	5’ – GCGTATGTGCTTCATGGCTG – 3’<br>5’ – TTAACACAGGCACCCGCC – 3’	Partial exon5 of *DEP1* gene
Dep1-MF<br>*Dep1-MR*	5’ – CAGATCGAAACTGTGCATAT – 3’<br>5’ – TACTAGAGCTACTTCACTCC – 3’	Complete exon5 and partial 3′ UTR of *DEP1* gene
M13F<br>*M13R*	5′-GTTTTCCCAGTCACGAC-3′<br>5′-AGCGGATAACAATTTCACACAGGA-3′	pGEM-T Easy vector

Table S4. p-distances between *DEP1* gene sequences from A, B and D genomes.

	DEP1-A T. aestivum (FAOM01374184)	***DEP1-A T. monococcum* (CS) (MF979621, MF979622**	***DEP1-A T. monococcum* (NS) (MF979623, MF979624)**	***DEP1-A T. durum* (CS) (MF979625)**	***DEP1-A T. durum* (NS) (MF979626)**	***DEP1-A T. compactum* (CS) (MF979627)**	***DEP1-A T. spelta* (NS) (MF979628**	predicted *DEP1-B T. aestivum* (CCYC011397742)	*DEP1-D T. aestivum* (FAOM01435944)	***DEP1-D T. compactum* (CS) (MF979629)**	***DEP1-D T. spelta* (NS) (MF979630)**
DEP1-A T. aestivum (FAOM01374184)											
***DEP1-A T. monococcum* (CS) (MF979621, MF979622)**	0.033										
***DEP1-A T. monococcum* (NS) (MF979623, MF979624)**	0.034	0.003									
***DEP1-A T. durum* (CS) (MF979625)**	0.004	0.033	0.034								
***DEP1-A T. durum* (NS) (MF979626)**	0.001	0.033	0.035	0.003							
***DEP1-A T. compactum* (CS) (MF979627)**	0.000	0.033	0.034	0.004	0.001						
***DEP1-A T. spelta* (NS) (MF979628)**	0.000	0.033	0.034	0.004	0.001	0.000					
predicted *DEP1-B T. aestivum* (CCYC011397742)	0.082	0.083	0.084	0.082	0.083	0.082	0.082				
DEP1-D T. aestivum (FAOM01435944)	0.079	0.084	0.085	0.079	0.079	0.079	0.079	0.046			
***DEP1-D T. compactum* (CS) (MF979629)**	0.081	0.087	0.088	0.081	0.082	0.082	0.082	0.049	0.002		
***DEP1-D T. spelta* (NS) (MF979630)**	0.081	0.087	0.088	0.081	0.081	0.081	0.081	0.048	0.003	0.002	

Table S5. p-distances between *VRN1* gene sequences from A, B and D genomes.

	VRN-A1 *T. aestivum* (KT696536)	*VRN-A1* *T. aestivum* (KT696535)	*VRN-A1* *T. aestivum* (JF965397)	*VRN-A1* *T. aestivum* (JF965396)	*VRN-A1* *T. aestivum* (JF965395)	*VRN-A1* *T. aestivum* (AY747601)	*VRN-A1* *T. aestivum* (AY747600)	*VRN-B1* *T. aestivum* (KR816809)	*VRN-B1* *T. aestivum* (AY747604)	*VRN-B1* *T. aestivum* (KR816810)	*VRN-D1* *T. aestivum* (AY747606)
VRN-A1 *T. aestivum* (KT696536)											
VRN-A1 *T. aestivum* (KT696535)	0.000										
VRN-A1 *T. aestivum* (JF965397)	0.002	0.002									
VRN-A1 *T. aestivum* (JF965396)	0.002	0.002	0.000								
VRN-A1 *T. aestivum* (JF965395)	0.002	0.002	0.002	0.002							
VRN-A1 *T. aestivum* (AY747601)	0.000	0.000	0.002	0.002	0.002						
VRN-A1 *T. aestivum* (AY747600)	0.000	0.000	0.002	0.002	0.002	0.000					
VRN-B1 *T. aestivum* (KR816809)	0.095	0.095	0.095	0.095	0.095	0.095	0.095				
VRN-B1 *T. aestivum* (AY747604)	0.096	0.096	0.097	0.097	0.097	0.096	0.096	0.003			
VRN-B1 *T. aestivum* (KR816810)	0.096	0.096	0.097	0.097	0.097	0.096	0.096	0.003	0.000		
VRN-D1 *T. aestivum* (AY747606)	0.100	0.100	0.100	0.100	0.100	0.100	0.100	0.038	0.040	0.040	

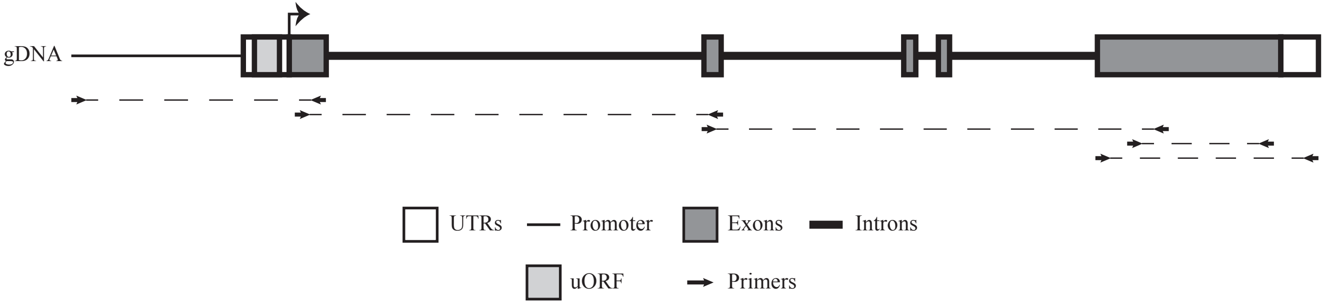

Figure S1. Schematic representation of the primer pairs positions.

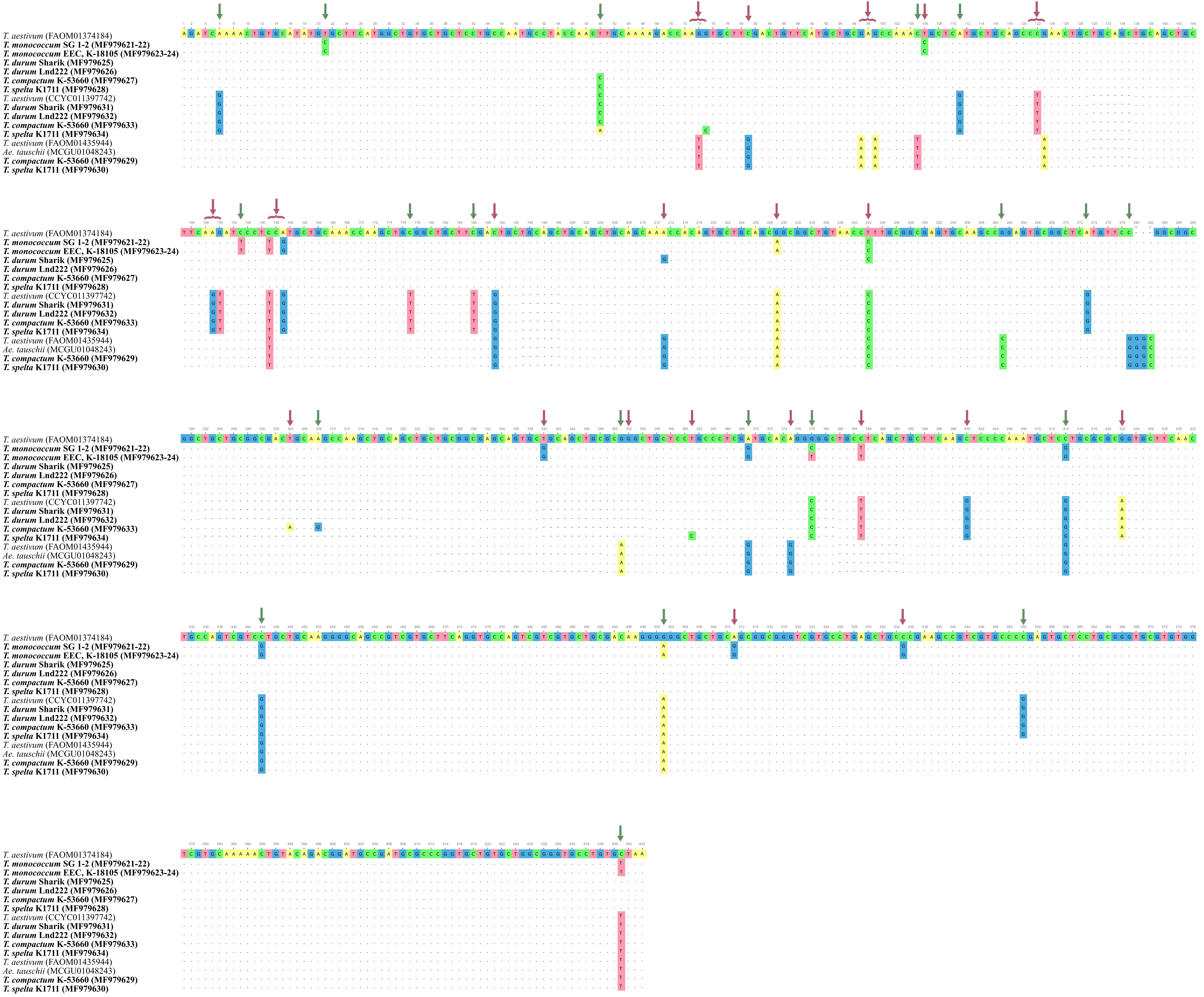

Figure S2. Nucleotide alignment of 5th exon of *DEP1* gene from different wheat species and *Ae. tauschii*. Sequence of *T. aestivum* (FAOM01374184) was used as a reference. Sequences obtained by experimental methods in the present study are marked in bold. Nucleotides, that match with reference sequence, are designated by dots. Nonsynonymous and synonymous amino acid substitutions are indicated by red and green arrows, respectively.
